# Advanced Thymoma and Advanced Thymic Carcinoma Treatment Practices in 2018–2022: A Nationwide Analysis in Japan

**DOI:** 10.1111/1759-7714.70367

**Published:** 2026-07-31

**Authors:** Junichi Ishigaki, Ryoko Rikitake, Hidenori Kage, Takahiro Higashi

**Affiliations:** ^1^ Department of Public Health and Health Policy, Graduate School of Medicine The University of Tokyo Tokyo Japan; ^2^ Department of Respiratory Medicine, Graduate School of Medicine The University of Tokyo Tokyo Japan; ^3^ Rare Cancer Center National Cancer Center Hospital Tokyo Japan; ^4^ Division of Health Services Research Institute for Cancer Control, National Cancer Center Tokyo Japan

**Keywords:** chemotherapy, epidemiologic studies, rare disease, thymic carcinoma, thymoma

## Abstract

**Introduction:**

Thymomas and thymic carcinomas are rare diseases with limited clinical evidence. This study aimed to investigate the clinical characteristics of and treatment practices for Stages III and IV thymoma or thymic carcinoma in Japan.

**Methods:**

We analyzed cases of Stage III and IV thymoma and thymic carcinoma diagnosed between 2018 and 2022 and recorded in a nationwide dataset linking the Hospital‐based Cancer Registry and the Diagnosis Procedure Combination survey. Treatment practices, including the use of perioperative chemotherapy, radiotherapy, and newer systemic therapies as well as the chemotherapy regimens, were evaluated.

**Results:**

Overall, 556 stage III thymoma, 312 stage III thymic carcinoma, 431 stage IV thymoma, and 976 stage IV thymic carcinoma patients were identified. Postoperative radiotherapy after complete resection was more common for stage III thymic carcinoma than for thymoma (34.1% vs. 10.7%), while perioperative chemotherapy was used more frequently for stage IV thymoma than for thymic carcinoma (preoperative: 15.5% vs. 3.7%; postoperative: 8.4% vs. 4.4%). For stage IV thymoma, adriamycin–cisplatin–vincristine–cyclophosphamide was the most common regimen (41.8%), whereas carboplatin–paclitaxel was predominantly used for stage IV thymic carcinoma (99.5%). The proportion of anthracycline‐based therapy for stage IV thymoma increased from 50.0% to 77.8%, while that of postoperative radiotherapy for stage III thymoma decreased from 11.1% to 4.1%.

**Conclusions:**

Evidence‐based treatment approaches for thymoma and thymic carcinoma have been widely adopted in Japan.

## Graphical abstract

In the field of rare diseases, where high‐quality evidence remains limited, this study demonstrated how controversial treatment practices are managed in real‐world settings on a nationwide scale. The proportion of anthracycline‐based chemotherapy for stage IV thymoma increased from 50.0% in 2018 to 77.8% in 2022.

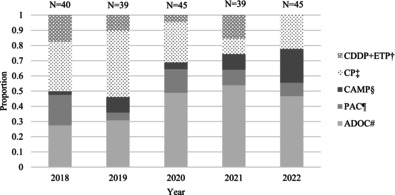

## 1 Introduction

Thymoma and thymic carcinoma are rare diseases, with thymoma incidence reported to be 0.44 to 0.68 cases per 100 000 population [1]. Thymomas are tumors arising from thymic epithelial cells, which are crucial in T‐lymphocyte maturation, and are characterized by the lack of cytologic atypia [2]. Thymic carcinoma is defined by the presence of cytologic atypia. Complete surgical resection remains the standard of care [1]. Nonetheless, patients with advanced tumors often require multimodal treatment strategies, including radiotherapy and chemotherapy. In unresectable disease cases, systemic chemotherapy, particularly platinum‐based regimens, is commonly used.

According to the Guidelines for Diagnosis and Treatment of Lung Cancer/Malignant Pleural Mesothelioma/Thymic Tumor 2025, combination therapy with cisplatin and anthracycline‐based agents is strongly recommended for patients with stage IV thymoma [1, 3]. Moreover, for those with stage IV thymic carcinoma, combination therapy with carboplatin and paclitaxel or amrubicin is weakly recommended. With regard to completely resected stage III thymoma, the guidelines state that evidence to clearly recommend postoperative radiotherapy is insufficient [1, 3]. In the National Comprehensive Cancer Network (NCCN) Guidelines, Version 2.2025, the NCCN Panel voted to designate cisplatin, doxorubicin, and cyclophosphamide (PAC) therapy as the preferred thymoma treatment [4] and carboplatin and paclitaxel (CP) therapy as the first‐line treatment for stage IV thymic carcinoma. The European Society for Medical Oncology guidelines for thymic epithelial tumors were updated in 2015 and have not been revised since [5].

Although the efficacy of individual chemotherapy regimens for thymic malignancies has been investigated previously [6, 7, 8, 9, 10, 11, 12, 13, 14, 15], most prior reports were derived from single institutions or limited case series. No prior studies have systematically evaluated the use of each regimen, and understanding of real‐world treatment practices remains limited.

Owing to the rarity of the disease, high‐quality evidence, including randomized controlled trials, remains limited; understanding current clinical practices is essential to inform future clinical decision‐making. Using large‐scale data from the Diagnosis Procedure Combination (DPC) survey and Hospital‐Based Cancer Registry (HBCR), the present study aimed to describe the management of stages III and IV thymoma or thymic carcinoma in relation to the recommendations in clinical practice guidelines, especially focusing on the use of perioperative chemotherapy, radiotherapy, and newer systemic therapies, as well as the chemotherapy regimens selected.

## 2 Methods

### 2.1 Data Source

We used a nationwide “Quality Indicator (QI) dataset” from 2018 to 2022, which included the DPC survey data and HBCR data in a linkable format for a quality‐of‐care monitoring project [16, 17]. Each patient was assigned a unique identifier within the QI dataset. The DPC survey collects health service utilization data, including surgeries, drug administration, laboratory tests, and radiotherapy, from hospitals that use the DPC‐based payment system for inpatient care and from hospitals preparing to introduce this system. The HBCR systematically collects and manages data on patients with cancer diagnosed and treated in hospitals. The registry records cancer type, site, histological classification, stage, date of diagnosis, and first‐course treatment. Stage was recorded according to the Tumor–Node–Metastasis classification and staging system of the Union for International Cancer Control [18]. HBCR data are collected from approximately 850 facilities, mainly designated cancer hospitals, and cover approximately 70% of all cancer cases in Japan [19, 20]. The QI dataset collects data from facilities in the HBCR that agree to participate in a quality‐of‐care study and covers approximately 60% of all cancer cases nationwide [19, 20].

### 2.2 Patients

We analyzed the data of patients diagnosed with thymoma or thymic carcinoma between January 2018 and December 2022. The dataset recorded staging at the time of initial treatment. We used the International Classification of Diseases Oncology 3rd edition First Revision (ICD‐O‐3.1) [21] and Second Revision (ICD‐O‐3.2) [22] topography codes C379, C381, C382, and C383 and histology codes 8580/3, 8581/3, 8582/3, 8583/3, 8584/3, and 8585/3 to identify thymoma cases [23]. Moreover, we implemented ICD‐O‐3.1 and ICD‐O‐3.2 topography codes C379, C381, C382, and C383 and histology codes 8070, 8123, 8082, 8140, 8260, 8200, 8144, 8560, 8023, 8430, 8310, 8033, 8980, 8020, and 8586 to identify thymic carcinoma cases [23]. We extracted information on patients with thymoma and thymic carcinoma from the database, including sex, age, site, histological type, admission and discharge dates, stage classification, surgical procedures, radiotherapy, and chemotherapy details.

### 2.3 Statistical Analysis

For stage III thymoma and thymic carcinoma, the focus was on the use of perioperative chemotherapy and radiotherapy, and for stage IV thymoma and thymic carcinoma we analyzed the use of perioperative chemotherapy and newer systemic therapies. We specifically focused on postoperative radiotherapy for stage III thymoma and chemotherapy regimens for stage IV thymoma and thymic carcinoma, considering both overall practice patterns and temporal trends.

Categorical variables were compared using the chi‐squared test or Fisher's exact test, as appropriate. Temporal trends in the proportion of treatment regimens were analyzed using the Cochran–Armitage trend test. All statistical analyses were performed using the Stata Version 17.0 software (Stata Corp, College Station, TX, USA). Statistical significance was set at *p* < 0.05.

### 2.4 Ethical Considerations

The study protocol was approved by the Institutional Review Boards (IRBs) of the National Cancer Centre in Japan (approval number: 2024‐374) and the University of Tokyo (approval number: 2025315NIe). We used a QI dataset after obtaining permission to use the data for secondary purposes. When patients were fewer than 10, their numbers were masked to ensure privacy protection.

## 3 Results

**FIGURE 1:**
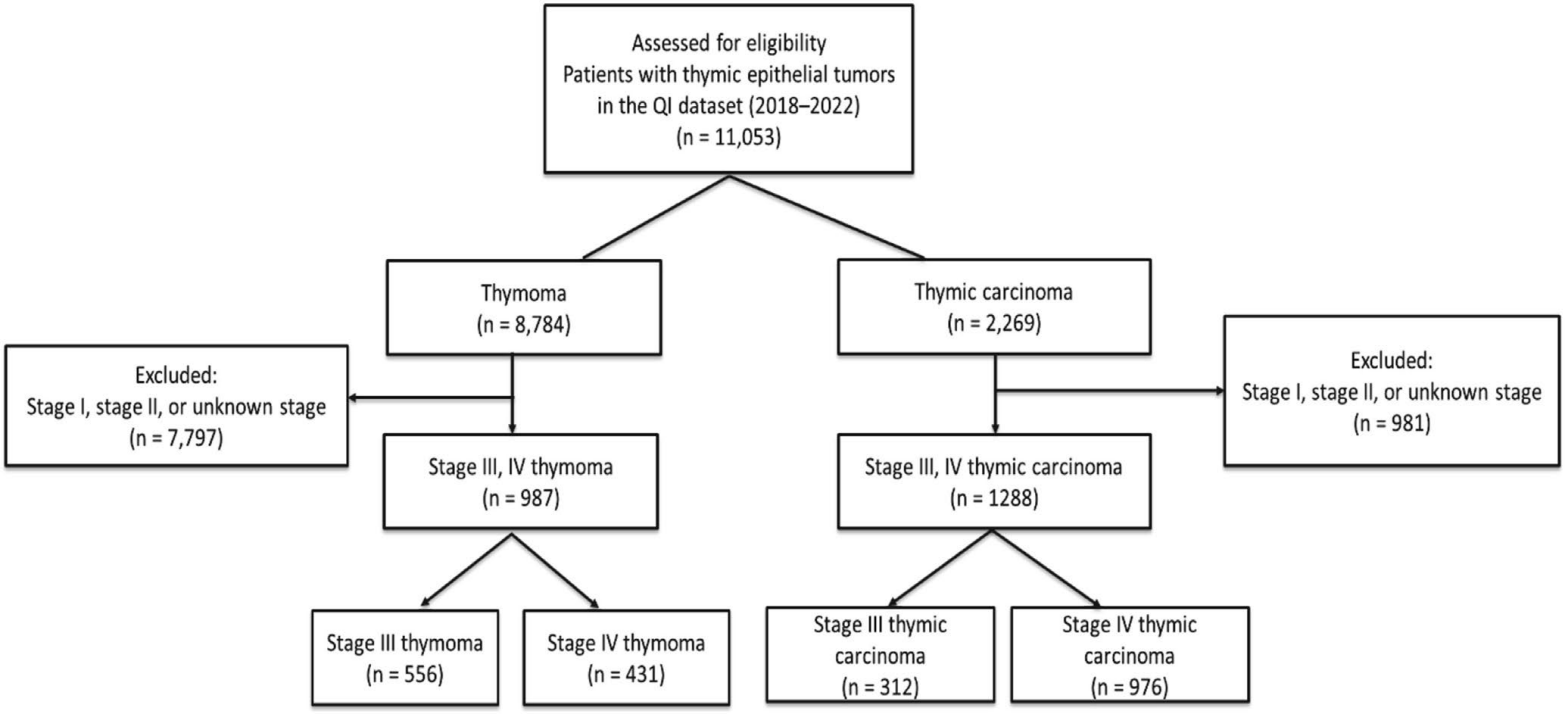
Patient selection: Patients were identified in the QI dataset between 2018 and 2022 and classified by histology and stage. The final disease‐stage cohorts included stage III thymoma, stage IV thymoma, stage III thymic carcinoma, and stage IV thymic carcinoma.

We extracted data from 8784 and 2269 patients with thymoma and thymic carcinoma, respectively, from the QI dataset between 2018 and 2022. Table 1 displays the baseline characteristics of patients. Table 2 show the characteristics of the histological types of thymoma and thymic carcinoma, respectively. Figure 1 illustrates the patient selection process. Among the 8784 patients with thymoma, 7797 with stage I, stage II, or unknown stages were excluded. From the total of 2269 patients with thymic carcinoma, 981 with stage I or II disease or unknown stages were excluded. Consequently, we identified 556 with stage III thymoma, and 312 with stage III thymic carcinoma, and 431 patients with stage IV thymoma, 976 with stage IV thymic carcinoma, from the QI dataset between 2018 and 2022.

**TABLE 1:** Characteristics of patients with thymoma and thymic carcinomas.

		Thymoma	Thymic carcinoma
		*N* (%)	*N* (%)
Sex	Male	4017 (45.7)	1458 (64.3)
	Female	4767 (54.3)	811 (35.7)
Age	< 40	510 (5.8)	46 (2.0)
	40–49	1225 (14.0)	164 (7.2)
	50–59	1775 (20.2)	353 (15.6)
	60–69	2145 (24.4)	675 (29.8)
	70–79	2473 (28.2)	790 (34.8)
	≧ 80	656 (7.5)	241 (10.6)
	Median	65	69
Stage	I	5889 (67.0)	596 (26.3)
	II	400 (4.6)	108 (4.8)
	III	556 (6.3)	312 (13.8)
	IV	431 (4.9)	976 (43.0)
	Unknown	1508 (17.2)	277 (12.2)
Total		8784 (100)	2269 (100)

**TABLE 2:** Histological types of (A) thymoma and (B) thymic carcinoma.

Pathology	*N* (%)
*(A) Thymoma*	
Type AB thymoma	2386 (27.2)
Type B2 thymoma	2152 (24.5)
Type B1 thymoma	1978 (22.5)
Type A thymoma	1032 (11.8)
Type B3 thymoma	846 (9.6)
Others	390 (4.4)
Total	8784 (100)
*(B) Thymic carcinoma*	
Squamous cell carcinoma	1699 (74.9)
Thymic carcinoma	447 (19.7)
Adenocarcinoma	64 (2.8)
Undifferentiated carcinoma	32 (1.4)
Others	27 (1.2)
Total	2269 (100)

For stage III thymoma, 375 (67.4%) and 84 (15.1%) of 556 patients underwent complete and incomplete resection, respectively, whereas 68 patients did not undergo surgery and surgical status was unknown for 29 patients. Preoperative and postoperative chemotherapy were administered to 66 (11.9%) and 33 (5.9%) patients, respectively. For stage III thymic carcinoma, 167 (53.5%) and 59 (18.9%) of 312 patients underwent complete and incomplete resection, respectively, whereas 80 patients did not undergo surgery and surgical status was unknown for 6 patients. Among patients with completely resected stage III disease, postoperative radiotherapy was administered to 40 of 375 patients (10.7%) with thymoma and 57 of 167 patients (34.1%) with thymic carcinoma. This result indicated a significantly higher frequency of postoperative radiotherapy for thymic carcinoma (*p* < 0.001).

**FIGURE 2:**
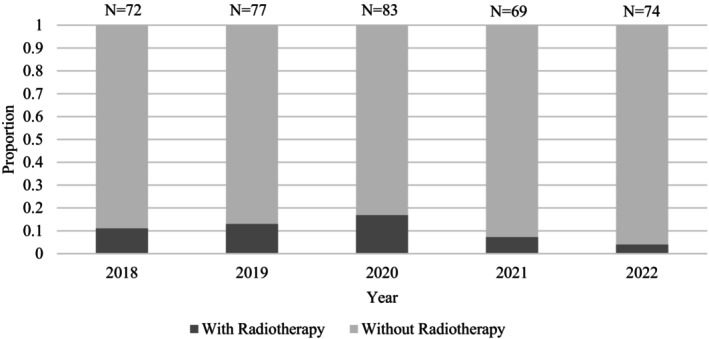
Proportion of patients receiving postoperative radiotherapy for completely resected stage III thymomas.

Among 556 patients with stage III thymomas, 375 (67.4%) underwent complete resection. Figure 2 displays the proportion of patients with completely resected stage III thymoma who underwent postoperative radiotherapy. Among these 375 patients, 40 (10.7%) received postoperative radiotherapy. The proportion of patients receiving postoperative radiotherapy fluctuated, with transient increases in some years; however, an overall decreasing trend was observed over the 5‐year period from 2018 to 2022. The rate declined from 11.1% in 2018 to 4.1% in 2022, although this trend was not statistically significant (*p* = 0.081). For stage IV thymoma, preoperative chemotherapy was administered to 67 of 431 patients (15.5%), whereas postoperative chemotherapy was administered to 36 patients (8.4%). Surgical resection was performed for 188 patients (43.6%). Target therapy was administered to 11 patients (2.6%), and fewer than 10 patients received immune therapy. For stage IV thymic carcinoma, preoperative and postoperative chemotherapy were administered to 36 (3.7%) and 43 (4.4%) of 976 patients, respectively. Target therapy was administered to 295 patients (30.2%), while immune therapy was administered to 65 patients (6.7%). Perioperative chemotherapy was more frequently used for stage IV thymoma than for stage IV thymic carcinoma (preoperative: 15.5% vs. 3.7%, *p* < 0.001; postoperative: 8.4% vs. 4.4%, *p* = 0.003). In contrast, target therapy was more frequently administered for stage IV thymic carcinoma than for stage IV thymoma (30.2% vs. 2.6%, *p* < 0.001).

**FIGURE 3:**
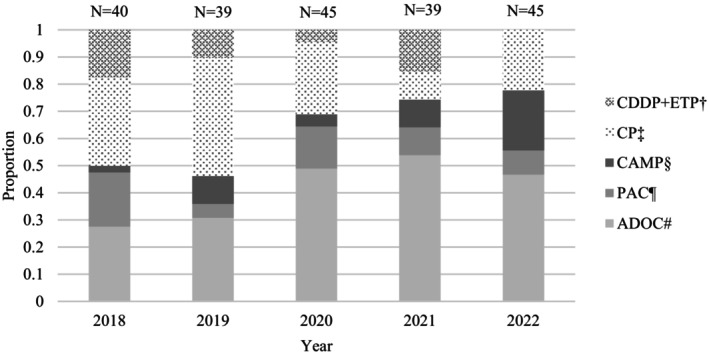
Proportion of chemotherapy regimens for stage IV thymoma: Cisplatin and etoposide; carboplatin and paclitaxel; cisplatin, doxorubicin, and methylprednisolone; cisplatin, doxorubicin, and cyclophosphamide; and doxorubicin, cisplatin, vincristine, and cyclophosphamide. ADOC, doxorubicin, cisplatin, vincristine, and cyclophosphamide; CAMP, cisplatin, doxorubicin, and methylprednisolone; CDDP+ETP, cisplatin and etoposide; CP, carboplatin and paclitaxel; PAC, cisplatin, doxorubicin, and cyclophosphamide.

**FIGURE 4:**
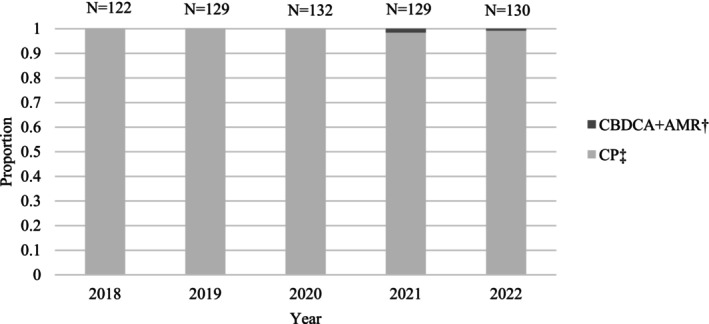
Proportion of chemotherapy regimens used for stage IV thymic carcinoma: Carboplatin and amrubicin, and carboplatin and paclitaxel. CBDCA+AMR, carboplatin and amrubicin; CP, carboplatin and paclitaxel.

In total, 208 of 431 patients (48.3%) with stage IV thymoma and 642 of 976 patients (65.8%) with stage IV thymic carcinoma received chemotherapy without surgical treatment. In patients with stage IV thymoma, the adriamycin, cisplatin, vincristine, and cyclophosphamide (ADOC) regimen was the most frequently administered (*n* = 87, 41.8%), followed by the CP regimen (*n* = 56, 26.9%). Figure 3 presents the proportion of anthracycline‐based combination therapies (ADOC, PAC, CAMP [cisplatin, doxorubicin, and methylprednisolone]) and non‐anthracycline regimens (CP, CDDP + ETP [cisplatin and etoposide]) for stage IV thymoma from 2018 to 2022. The proportion of anthracycline‐based regimens increased over time from 50.0% in 2018 to 77.8% in 2022 (Cochran–Armitage test for trend: *p* < 0.001). The proportions of chemotherapy regimens for stage IV thymic carcinoma are summarized in Figure 4. Nearly all patients with stage IV thymic carcinoma received the CP regimen (*n* = 639, 99.5%), and no clear temporal changes were observed over the study period.

## 4 Discussion

In this study, we described the treatment practices for advanced thymoma and thymic carcinoma using nationwide data from Japan. To our knowledge, this is the first study to examine such treatment practices in detail. Specifically, we separately evaluated postoperative radiotherapy for completely resected stage III thymoma, chemotherapy regimens for stage IV thymoma, and chemotherapy regimens for stage IV thymic carcinoma, because these are all clinically important conditions with variable and controversial treatment practices.

For completely resected stage III thymoma, current guidelines state that evidence to clearly recommend postoperative radiotherapy is inadequate. In contrast, postoperative radiotherapy is recommended for incompletely resected stage III thymoma and stage III thymic carcinoma. Therefore, we did not focus on incompletely resected stage III thymoma and stage III thymic carcinoma.

Among patients with completely resected stage III thymomas, 10.7% received postoperative radiotherapy as part of their initial treatment. The effectiveness of postoperative radiotherapy for completely resected stage III thymomas remains controversial, as findings have been inconsistent [24, 25, 26, 27, 28, 29, 30, 31]. Clinical practice guidelines for lung cancer indicate insufficient evidence to recommend postoperative radiotherapy for completely resected stage III thymoma [1, 3]. The 2016 Japanese guidelines already stated that postoperative radiotherapy was not recommended [32], and this stance remained unchanged in subsequent revisions [1, 33]. Consistent with these guidelines, our study found no increase in the use of postoperative radiotherapy.

For patients with stage IV thymoma, combination therapy with cisplatin and anthracycline‐based agents is strongly recommended; however, it is highly toxic. For patients with stage IV thymic carcinoma, combination therapy with carboplatin and paclitaxel or amrubicin is weakly recommended; however, the implementation rates of individual regimens remain unclear. Among patients with stage IV thymoma, the ADOC regimen was most frequently used in 2022, whereas only a minority was administered ADOC in 2018. The use of anthracycline‐based combination therapies for stage IV thymoma has recently increased. The CP regimen is the most commonly administered regimen for stage IV thymic carcinomas.

For advanced thymoma, platinum‐based regimens with anthracyclines have demonstrated higher response rates than have non‐anthracycline regimens in prior studies [6, 7, 8, 9, 10, 11, 12, 13]. Nevertheless, the available evidence is limited to Phase II studies and retrospective analyses. Ideally, phase III trials would provide more definitive evidence; however, it is not easy to conduct such studies for rare cancers. Therefore, real‐world data, including progression‐free survival and overall survival, are warranted to fill in the evidence gap regarding treatment effectiveness. In this analysis, treatment outcomes were not evaluated because DPC data do not have accurate mortality or recurrence data. According to the Guidelines for Diagnosis and Treatment of the Lung Cancer/Malignant Pleural Mesothelioma/Thymic Tumors 2025, in stage IV thymoma, combination therapy with cisplatin and anthracycline‐based agents is recommended rather than regimens that do not include anthracyclines [1, 3]. This recommendation was explicitly stated in the 2018 revision of Japanese guidelines [33]. In the 2016 edition, the guidelines took a cautious stance, noting that “although scientific evidence was insufficient, this treatment may be considered” [32]. Therefore, the increased use of anthracycline‐based regimens after 2018 likely reflects the impact of the guideline revision. In our study, the use of anthracycline‐based regimens for stage IV thymoma increased over the study period, suggesting greater adherence to guideline recommendations. Nonetheless, the increase was not as pronounced as that with the CP regimen in thymic carcinoma, despite the weaker recommendation for the CP regimen. A potential explanation is that anthracycline‐based regimens are not typically part of routine thoracic oncology practice. Furthermore, the evidence supporting anthracycline‐based regimens for thymoma is limited to several phase II studies and remains insufficiently robust [6, 7, 8, 9, 10, 11, 12, 13]. Thus, determining whether anthracycline‐based regimens should be broadly adopted for thymoma treatment requires additional evidence. The preference for ADOC over PAC may be explained by the difference in reported response rates. ADOC demonstrated a higher overall response rate (ORR) (91.8%) and complete response rate (CR) (43.8%) [6], whereas PAC showed lower efficacy, with an ORR of 50% and a CR of 10% [7]. Chemotherapy was implemented for 48.3% patients. The QI dataset did not contain information relevant to treatment decision‐making such as performance status and comorbidities; therefore, we were unable to assess the reasons for the relatively low chemotherapy utilization.

In contrast, current guidelines do not recommend anthracycline‐based combination therapy for stage IV thymic carcinoma, but weakly recommend combination therapy with carboplatin, paclitaxel, or amrubicin [1, 3]. The NCCN guidelines state that the CP regimen is preferred as the first‐line therapy [4]. Here, nearly all patients received the CP regimen, possibly because thoracic oncologists were comfortable with it given its widespread use in lung cancer. Target therapy was used for 30.2% of patients. The QI dataset captures treatments administered during the observation period and does not allow reliable differentiation between first‐line and subsequent lines of therapy. Therefore, some patients included in this analysis may have received molecular targeted therapy during later lines of treatment after first‐line treatment initiation.

This study had some limitations. First, although we used a nationwide dataset, it primarily included designated cancer hospitals and may not have fully captured treatment practices at smaller or non‐participating institutions. Conversely, designated cancer care hospitals are key to the management of thymoma and thymic carcinoma in Japan, making them appropriate for capturing standard treatment trends in real‐world practice. Moreover, as the dataset covers approximately 60% cases nationwide, its representativeness is sufficiently high [19, 20]. Second, our DPC survey data are available only for up to 2 years after registration in the HBCR, limiting the ability to capture long‐term treatment courses and subsequent treatment. Nevertheless, this study primarily aimed to clarify treatment practices in the early treatment phases rather than to evaluate long‐term outcomes; thus, this limitation is unlikely to have substantially affected the study objectives. Third, treatment data were obtained only from hospitals that provided the initial treatment, and treatments delivered in other facilities were not captured; therefore, the effectiveness of radiation therapy could not be fully evaluated. Nonetheless, all designated cancer care hospitals have radiation equipment as a condition for designation; accordingly, we expect that only a minority of patients received radiation therapy at other facilities. Finally, detailed clinical information such as performance status, biomarker profiles, and treatment response was not available. The QI dataset used in this study does not contain detailed outcome data; therefore, we were unable to evaluate the efficacy of each chemotherapy regimen in the present analysis. Although individual clinical backgrounds could not be assessed, this study captured nationwide trends using large‐scale data and described treatment practices for rare cancers on this scale, which is a significant strength.

In conclusion, using a nationwide dataset, we provided detailed information on the clinical features of thymoma and thymic carcinoma in Japan. This study revealed that while treatments for thymic carcinoma are largely consistent with guideline recommendations, some patients with thymoma do not receive the recommended anthracycline‐based regimens. Further evidence is required to determine whether anthracycline‐based regimens should be adopted more broadly for thymomas.

## Author Contributions

**Junichi Ishigaki:** conceptualization, methodology, investigation, writing – original draft. **Takahiro Higashi:** conceptualization, methodology, project administration, supervision, resources, writing – review and editing. **Hidenori Kage:** Supervision, Writing – Review & Editing. **Ryoko Rikitake:** methodology, supervision, writing – review and editing.

## Funding

The authors have nothing to report.

## Consent

The need for patient consent was waived because of the retrospective nature of the study and the use of anonymized data.

## Conflicts of Interest

The authors declare no conflicts of interest.

## Data Availability

The authors have nothing to report.
